# Engagement Book

**Published:** 1881-01

**Authors:** 


### ENGAGEMENT BOOK.

The Dentist's Memorandum and Engagement Book recently issued by the Editor of the Register, may be obtained at the Dental Depots.

It will be found to meet all the requirements of the dentist in this direction.

